# Why we do need a new gold open access journal called “Brain, behavior and immunity - Health”

**DOI:** 10.1016/j.bbih.2019.100001

**Published:** 2019-11-18

**Authors:** Annamaria Cattaneo, Ebrahim Haroon, Kuan-Pin Su, Carmine M. Pariante

**Affiliations:** Head of the Biological Psychiatry Laboratory, IRCCS Centre, Fatebenefratelli Brescia, Italy; Department of Psychiatry & Behavioural Sciences, Emory University, Atlanta, GA, USA; Department of Psychiatry & Mind-Body Interface Laboratory (MBI-Lab), China Medical University Hospital, Taichung, Taiwan; Institute of Psychiatry, Psychology and Neuroscience, King’s College London, London, UK

**Keywords:** Brain, behavior, Immunity, Psychoneuroimmunology, Neuroimmunology, Immunopsychiatry, Journal, Research

## Abstract

This Editorial discusses the missions and scope of the new Gold Open Access journal “Brain, Behavior and Immunity (BBI) – Health” and how it complements the activity of the established BBI journal.

*This Viewpoint is published concomitantly in both Brain Behavior, & Immunity and the new journal, Brain, Behavior & Immunity-Health, to reach the widest possible audience*.

Where do scientific papers go when they are rejected from Brain, Behavior & Immunity (BBI)?

If you are reading this editorial, you probably are familiar with the missions and scope of BBI (Kelley, 2017; Pariante, 2018; Pariante et al., 2019). Dedicated to studies dealing with behavioural, neural, endocrine, and immune system interactions in humans and animals, BBI is an international, interdisciplinary journal devoted to promoting original research in neuroscience, immunology, integrative physiology, behavioural biology, psychiatry, psychology, and clinical medicine, and is inclusive of research at the molecular, cellular, social, and whole organism level. The term map of all the papers published in BBI between 2013 and 2017 (Fig. 1) shows the breadth and depths of the topics covered by the journal (most impactful topics in terms of citations in red, least impactful topics in blue).

**Fig. 1:**
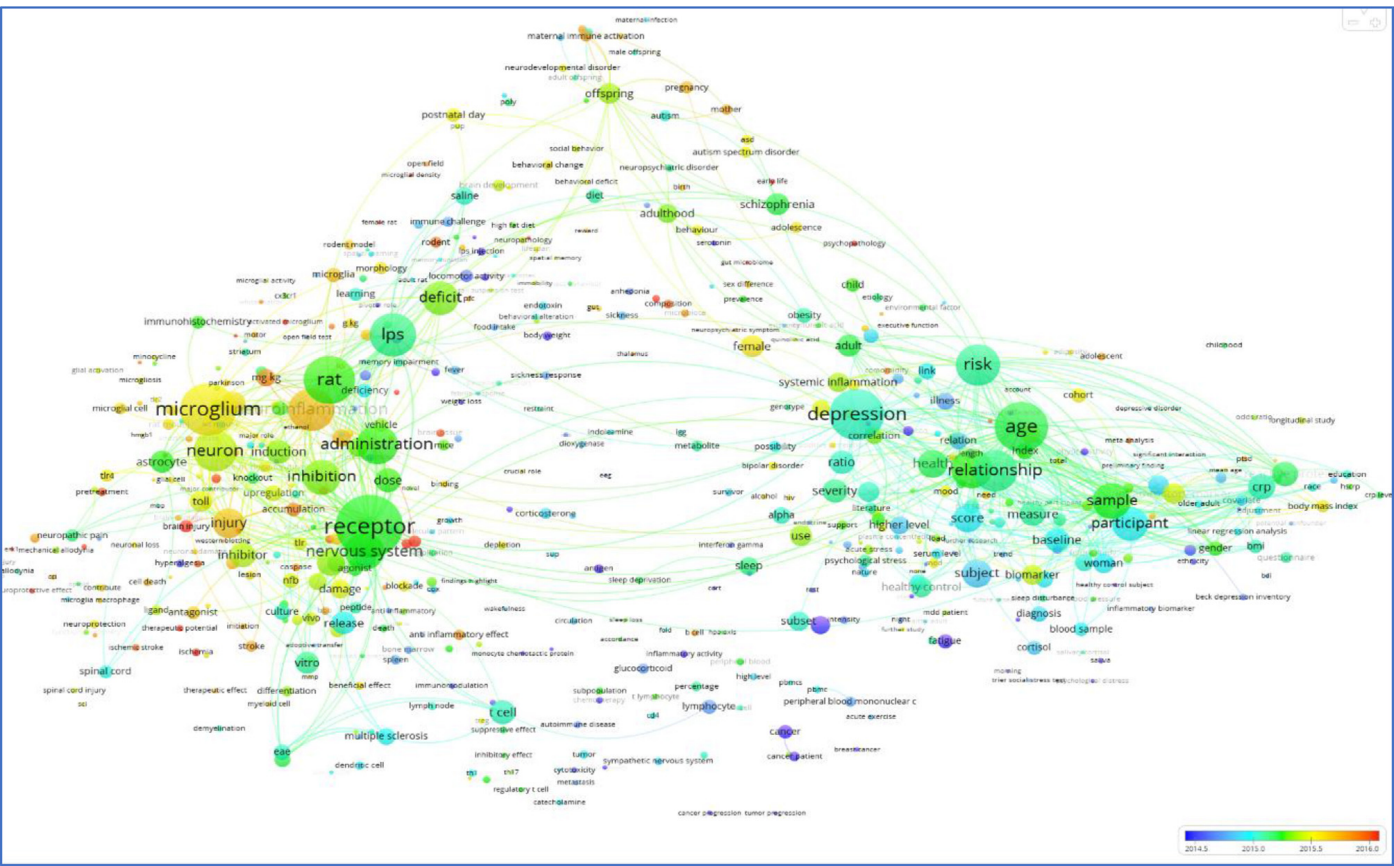
The term map of all the papers published in BBI between 2013 and 2017 shows the breadth and depths of the topics covered by the journal (most impactful topics in terms of citations in red, least impactful topics in blue).

With a healthy impact factor stably hovering around 6 over the last few years, BBI receives approximately 1000 submission per year – and it rejects around 750 of them. Many of these are good papers, but are incompatible with the missions and scope of BBI. We have decided to capture at least some of the rejected papers in a sister journal, named *BBI-Health*.

There are many reasons why a paper is unsuitable from BBI, and many of the rejected papers are valuable studies that end up being published in other good journals. An internal study conducted by Elsevier has tracked down papers rejected from BBI in 2013–2017, which then went on to be published in other scientific journals. Elsevier used the title and the authors’ names to track each rejected paper, to see if they got published and, if so, where and when, and how each paper performed in terms of citations. Inevitably, some papers would have been untraceable because the title was changed, the data split or merged within other publications, or the authors had simply moved on and not published it. But, of those that were subsequently published and traceable, most were eventually published in quality journals. Ironically, incorporation of comments provided by the associate editors and reviewers of BBI may have made these submissions more competitive for other journals, much to their benefit.

So, why were these papers rejected by BBI? Within BBI, there is an emphasis on studies that have a mechanistic component (i.e., try to explain what process underpins the results) and that have both biological and behavioural data (including some measure of immunity in its broader sense). So, papers that are scientifically sound that tend to be more descriptive without focussing on mechanisms; or test hypotheses that have been extensively investigated before for additional insights; or lack a behavioural outcome measure; or present conclusions that are considered too preliminary - are all considered unsuitable for BBI. For example, some papers are clinical studies using case-control comparisons of small samples or attempting to draw novel insights by measuring descriptive biomarkers that have been investigated before – often in the same types of clinical groups. And some are pre-clinical studies, like animal investigations testing or repurposing a me-too drug that affects a well-known mechanism for novel uses, with or without behavioural, clinical or functional endpoints. In summary, several of these rejections result from incompatibilities with the higher threshold for acceptance to BBI, which no doubt has led to its success. Yet, these papers could be helpful in moving research on psychoneuroimmunology forward, and often have clinical relevance.

BBI-Health has been launched in order to broaden the canvas of research on psychoneuroimmunology by capturing at least some of these good-quality-yet-rejected papers. By doing so, BBI-Health would also leverage the time and effort invested by the BBI editors and reviewers in reshaping rejected publications into competitive submissions – but no longer for other journals. BBI-Health will allow the transfer of papers from BBI to BBI-Health, either before peer-review (if the paper is clearly deemed unsuitable for BBI yet of sufficient quality) or after peer-review (if the referees highlight concerns akin to what we have described above). The good news is that authors will be able to revise their paper based on the referees’ comments even if the paper is rejected by BBI and transferred to BBI-Health, thus minimising delays in the editorial process. BBI’s referees too, hopefully, will find their review efforts put to good use - even if the paper is rejected from BBI. Of course, the editorial team will ensure that their suggested changes are integrated in the revised version before submission to BBI-Health.

But BBI-Health is not, and will not be, just about capturing rejected papers – it will be about *broadening the scope and expanding the readership* of both our journals, and about drawing more investigators into our field of research. Over time, we are confident that the clinical focus of the journal (when compared with BBI) will become clear to the broader family of readers and scientists, many of whom may or may not be members of the affiliated society, the Psychoneuroimmunology Research Society. We are immediately inviting direct submission to BBI-Health for papers that fulfil the broader criteria described above of strong emphasis on translation and clinical, rather than mechanistic, relevance.

Moreover, BBI-Health will publish paper formats that are clinically very relevant but yet are simply not accepted in BBI: research protocols of clinical studies and clinical trials; case reports or case series at the interface between psychology, psychosomatics, immunology, psychiatry and neurology; realist reviews, illustrating the method for implementation research; and papers discussing policy, including ethical, health and cultural implications of research in psychoneuroimmunology. BBI-Health will be broader – not narrower – than its older sister, BBI.

Finally, there is an ambition that BBI-Health will be an open-access, high-quality journal that will fulfil the criteria of the new ‘open access’ policy detailed in ‘Plan S’ and endorsed by a number of funders in the cOAlition S, including the Wellcome Trust, UK Research and Innovation, the European Research Council and the Bill & Melinda Gates Foundation who may eventually stop funding open-access publications in hybrid journals. If or when this happens, the community of scientists interested in psychoneuroimmunology and immunopsychiatry will be ready with a dedicated journal.

We are already thinking at what data we can publish in BBI-Health. Are you?
